# The facilitating factors and barriers encountered in the adoption of a humanized birth care approach in a highly specialized university affiliated hospital

**DOI:** 10.1186/1472-6874-11-53

**Published:** 2011-11-25

**Authors:** Roxana Behruzi, Marie Hatem, Lise Goulet, William Fraser

**Affiliations:** 1 McGill University, Faculty of Medicine, Department of Family Medicine, Montreal, Canada; 2 Université de Montréal, Faculty of Medicine, Department of Social and Preventive Medicine, Montreal, Canada; 3 Université de Montréal, Faculty of Medicine, Department of Obstetrics and Gynecology, Montreal, Canada

## Abstract

**Background:**

Considering the fact that a significant proportion of high-risk pregnancies are currently referred to tertiary level hospitals; and that a large proportion of low obstetric risk women still seek care in these hospitals, it is important to explore the factors that influence the childbirth experience in these hospitals, particularly, the concept of humanized birth care.

The aim of this study was to explore the organizational and cultural factors, which act as barriers or facilitators in the provision of humanized obstetrical care in a highly specialized, university-affiliated hospital in Quebec province, in Canada.

**Methods:**

A single case study design was chosen. The study sample included 17 professionals and administrators from different disciplines, and 157 women who gave birth in the hospital during the study. The data was collected through semi-structured interviews, field notes, participant observations, a self-administered questionnaire, documents, and archives. Both descriptive and qualitative deductive content analyses were performed and ethical considerations were respected.

**Results:**

Both external and internal dimensions of a highly specialized hospital can facilitate or be a barrier to the humanization of birth care practices in such institutions, whether independently, or altogether. The greatest facilitating factors found were: caring and family- centered model of care, professionals' and administrators' ambient for the provision of humanized birth care besides the medical interventional care which is tailored to improve safety, assurance, and comfort for women and their children, facilities to provide a pain-free birth, companionship and visiting rules, dealing with the patients' spiritual and religious beliefs. The most cited barriers were: the shortage of health care professionals, the lack of sufficient communication among the professionals, the stakeholders' desire for specialization rather than humanization, over estimation of medical performance, finally the training environment of the hospital leading to the presence of too many health care professionals, and consequently, a lack of privacy and continuity of care.

**Conclusion:**

The argument of medical intervention and technology at birth being an opposing factor to the humanization of birth was not seen to be an issue in the studied highly specialized university affiliated hospital.

## Background

Childbirth is both a social and cultural phenomenon with political implications. It has changed dramatically in the twentieth century, both in developed and developing countries [1-4]. Since the 1980s, increases in the rates of medical interventions at birth, such as the use of epidural analgesia, and cesarean sections, have also raised concerns not only among feminist activists with regards to women's right to have a 'natural' or 'normal' birth, but also in the Society of Obstetricians and Gynecologists of Canada [1,5-7].

In 2008-2009, the total caesarean section rates in Canada were 26.3% [8]. Moreover, about two-thirds (69.0%) of all vaginal deliveries in Quebec, and 60% in Ontario, were preceded by epidural analgesia. Electronic Fetal Monitoring (EFM), which was originally designed for high-risk pregnancy, is used in up to 90% of laboring women in recent years in Canada [9,10], despite the lack of evidence of its benefits. The total induction rate in Canada ranged from 20.7 to 23.7 per 100 hospital deliveries [11]. In North America, preterm birth rates increased from 6% in the early 1980s to 8% in more recent years, at least part of the increase is the result of iatrogenecity [11,12].

Noticeably, women's request for caesarean section or for a pain-free birth seems to have played an important role in the increase of caesarean and epidural analgesia's rates in past years [5,13-15]. Beckett (2005) argued that many women who choose cesarean section or epidural analgesia, may not be aware of the side effects of these interventions and are prone to make choices based on insufficient information [5]. Moreover, women who choose hospital births and obstetric technology seem to do it out of concern for their baby's safety [14]. According to Davis-Floyd (1994), American women who opt for the highest level of medical technology at birth, view these interventions as a form of control and empowerment over birth, rather than a loss of autonomy over it [16]. On the other hand, the attitude and beliefs of the maternity care professionals towards childbirth and the way they see birth may have the greatest impact on childbirth care, specifically humanized birth care. Klein and colleagues' study on the attitudes of Canadian maternity care practitioners towards labour and birth [17] showed that the family physicians who practice intrapartum care, as well as nurses, had intermediate score towards using obstetrical technology; however, obstetricians and family physicians who provide only antenatal care had more positive attitudes towards technology. Another study by Klein et al [18] showed that younger obstetricians were more pro-technology in normal birth, including routine epidural analgesia, and less supportive of women's control on their own childbirth.

The preliminary findings of our study revealed that the humanization of birth in a highly specialized university-affiliated hospital is in fact perceived through a different set of key concepts, these being: *security or safety, reassurance*, and *comfort*. These concepts were actualized by taking into account access to modern technology, high levels of monitoring, and professional expertise. In the studied institution, *personalized care*, *women's advocacy, companionship*, *reception of continued physical and psychological support by health care providers in a family-centered context*, were shown to be the best advocates of humanized birth care.

Considering the fact that a significant proportion of high-risk pregnancies currently receive care in highly specialized hospitals; and that an important number of low risk women also seeks care in these hospitals, it becomes important to understand and explore the factors which may influence the childbirth experience in these hospitals, particularly, the concept of humanized birth care. The humanization of care in a specialized hospital cannot be achieved if the external organizational factors, or its internal components are conceived separately [19].

We used the organizational culture model introduced by Allaire and Firsirotu (1984), in order to explore which of the external factors (history, society, contingencies) and the internal components of the institution (structure, culture, individuals) could act as barriers or facilitators to the humanization of birth practice in such hospitals. The authors considered the key concepts of humanization of birth as mentioned above.

The main research question was: in a specialized and university affiliated hospital, which internal and external components of the institution act as facilitators or barriers for adopting a humanized childbirthing care?

## Methods

### Study Design, Setting

The design is a case study involving a single hospital. The selected case is a highly specialized university-affiliated hospital in Montreal, Quebec, Canada, with 450 beds, including 30 beds at the Intensive Care Unit. Nearly 3900 births take place in the hospital every year, and the rate of caesarian section is approximately %29 of all deliveries [20]. The reputation of the hospital in providing care for women at high-obstetric-risk made it a preferred tertiary level referral centre for high-risk pregnancy patients (%40), preterm and very preterm births as well as sick children in the province of Quebec. Whilst many of women which had been referred to this institution were labeled as being at high-obstetric-risk, and thus needing specialized attention and intervention, there were the majority of women who were cared for, at the same hospital, but did not suffer from complications described as being at high-obstetric-risk.

The case study is composed of three key stakeholder groups: 1) administrators, 2) professionals, and 3) women and families.

The study sample consists of: 1) eleven professionals from different disciplines including: nurses, obstetricians, pediatricians, and anesthetists, 2) six administrators from different hierarchical levels of the hospital, including: executive client-program management, quality and risk-assessment management, management of clinical services, and nursing care management, and 3) a total of 157 women who gave birth in the center during the study period

The sample size of women was calculated to reach a confidence level of 0.95, a 2-sided interval, a standard deviation of 0.6 from a previous study (De Koninck, 2001), and a distance from mean to limit of 0.1 for a number of 139 participants. To cover the probability of drop outs, the total sample for this study was calculated to be 180 women.

The professional and administrative participants were chosen intentionally from different disciplines, and with varied levels of work experience. The women participants in the questionnaire group were chosen randomly from the total sample. Ten women were recruited to participate in the interviews with a broad diversity in pregnancy and delivery types.

For women, the inclusion criteria were as follows: at least 18 years- old, and able to speak, read and write in French or English (necessary for completing the questionnaire). They had to be within 24 to 48 hours postpartum, they had to have given birth in the hospital; and finally, they had to give their consent in order to participate. Exclusion criteria included women with intrauterine death -this was due to the fact that such a condition may influence the childbirth experience.

### Data Collection

Data was collected through: in-depth, open-ended, semi-structural interviews; field notes; participant observations; a self-administered questionnaire, documents, and archives. This variety of data sources allowed the triangulation of the data from the mentioned sources, and thus allowed to obtain information on the individuals' behavior, not just their stated attitudes.

The interviews were conducted in French and lasted between forty and ninety minutes. The interviews were continued until saturation of data [21]. All interviews with the women participants were voice recorded and conducted, by the primary author, in the women's postpartum hospital room. An interview guide was prepared based on the conceptual framework and literature review. This guide had initially been pre-tested and validated before being used through separate interviews with two professional nurses and two women in birthing centres. The interviews were later translated into English for publication

The self-administered questionnaire that we used had been developed in the context of a study that assessed midwifery practice in Quebec, comparing it to the standard obstetrical care provided in the province [22]. The questionnaire was adapted for the needs of the present study and was written in both English and French. The questionnaire comprised four sections and ninety-four multiple-choice and open-ended questions. The questions covered the topics of maternity experience, health-related consultation habits, the pregnancy, and delivery and early-postpartum experience. Finally, the questionnaire also contained some additional personal and socio-demographic questions. The reliability of the questionnaire have been assessed by Cronbach's Alphas; its values ranged from 0.71 to 0.93 [22].

Several activities were also carried out in our study in order to maximize the validity and reliability of the qualitative findings. These included methods: obtaining coefficient reliabilities (≥ 80), triangulation of data, ensuring referential adequacy, persistent observation, and prolonged engagement [23,24].

Ethics approval was obtained from the *Health Research Ethics Board of Hospitals affiliated with the Université de Montréal*. Informed consent was obtained from all the voluntary participants. The women agreed to allow the investigator be an observer during their labour and delivery, and in the early postpartum period. The women were informed that withdrawal from the study was possible at any time, that they had the right to refuse to answer any of the questions, as well as the fact that participating in the study would not in any way impact on the care to be received. Regarding data confidentiality purposes, the investigator used a code instead of the participants' name on the transcripts.

The data-collection period for this study spanned from October 2007 to March 2008, and it continued until a sufficiently rich description of the concept under study was achieved [21].

### Data Analysis

#### Qualitative Data Analysis

In all, twenty-seven recordings were transcribed *verbatim *and checked for accuracy, then entered into the QDA Miner qualitative software (Package Version 3.2.3). The field notes gathered from the field visits, the observation sheets, and the archival and administrative documents were also entered into the same software. All transcripts were coded into their distinctive categories, and a deductive content analysis was subsequently performed. This deductive approach aimed to validate and build upon the conceptual framework and theory used for this study. Thus, initial coding began with the external and internal factors mentioned in Allaire's and Firsirotu's organizational culture theory, as well as some relevant previous research findings regarding the concept under study. Then, the investigator immersed herself in the data and allowed the themes and categories to emerge from the data [25]. The data matrices were used to enable comparisons. A sample of matrices of the study shown in the data matrices were used to enable systematic comparison [26].

#### Quantitative Analysis of Data

The concept of *humanized care *as identified through the questionnaire's data means that the care has been modified to make it more in conformity with a certain philosophy and it was seen as being: *'care which is adapted to women's needs, that reflects a trust in the woman's capabilities, that gives control to women over decisions and choices *'. The concept of *continuity of care *was assessed as being: *'the consistency in the content of follow up, such as: information, advice, explanations, etc*; and having '*no interruption in the care received e.g. different caregivers are seen; and care is a shared approach'*.

*Descriptive statistics *(means and standard deviations for continuous variables and proportions of the categorical variables) were used to summarize the responses collected in the self-administered questionnaires. Special attention was paid to the description of the quality and quantity of services received in the hospital, obstetrical interventions and neonatal outcomes, as well as the woman's overall satisfaction with her birthing experience, and the control they thought they had over it. All statistical analyses were done using the SPSS software *(version 16)*.

## Results

The mean age of the participating professionals' was 44 years, (range -23 to 56). The level of education for these participants was as follows: Bachelor's degree (4), College diploma (1), Masters in nursing (1), MD (3), MD and PhD (2). The mean age of the administrators was 49, (range - 38 to 60 years). Of these, four had a Masters in Science, one a Bachelor's degree, and one a DES in Health care administration. Two of the administrators had a background in nursing. A total of 157 women participated in the study. Of these, 58 (36.9%) had high-risk pregnancies The mean age of the parturient women was 31, (range 15 to 46 years). Most of The women, 83 (52.9%) were from the French-speaking Canadian citizens, 95 (60.5%) had a university level of education. Most women (111; 70.7%) were married and had annual family income equal or more than $65,000 (41; 4%). The socio-demographic and some childbirth characteristics of women participants are shown in table 1.

**Table 1:** Socio-demographic and childbirth characteristics of women participant

Characteristics		N = 157 (%)	Characteristics		N = 157 (%)
**Age**	Minimum	15	**Mode of Delivery**	Vaginal	102(65,0)
	Maximum	46		Caesarean section	48(30,60)
	Mean	31		Operational vaginal delivery	7(4,5)
					
**Nationality**	American Citizen	18(11,5)	**Reason for Caesarean**	Failure in progress of labour	12
	Canadian French Citizen	83(13,4)		Planned caesarean	6
	Canadian English Citizen	3(1,9)		FHR Abnormality	8
	Canadian new immigrant	24(24)		Previous C-section	11
	European Citizen	8(5,1)		Breech	5
	South America, Asia, Africa	21(13,4)		Medical indication in mother	6
					
**Education**	Primary School	2(1,3)	**Epidural Analgesia**	No	62(39,5)
	Secondary	20(12,7)		Yes	95(60,5)
	College	40(25,5)			
	University/college	95(60,5)			
					
**Marital status**	Married	111(70,7)	**Electronic Foetal Monitoring (EFM)**	Yes	154(98,1)
	Single	8(5,1)		No	3(1,9)
	Conjoin	36(22,9)			
	Divorced	2(1,3)			
					
**Job**	Yes	102(65,0)	**Onset of Labour**	Not started	20(12,7)
	No	55(35,0)		Spontaneous	74(47,1)
				Induced	63(40,1)
					
**Family annual income**	Less than 20 000 $	15(9,6)	**Women's position during delivery**	Lying down	114(72,6)
	20 000 $ to 34 999 $	27(17,2)		In a semi-reclined position	41(26,1)
	35 000 $ to 49 999 $	20(12,7)		In a squatting position	1(0,6)
	50 000 $ to 64 999 $	29(18,5)		Other	1(0,6)
	Over 65000 $	65(41,4)			
					
**Number of pregnancies**	≤2	95(60,5)	**The number of care providers during labour and delivery**	1-2	52
	3-4	52(33,1)		3-4	67
	≥5	10(6,4)		5≥	38
					
**Women attended in the prenatal meetings or classes**	Yes	75(47.8)	**Complication during labour**	No	142(90,4)
	No	82(52,2)		Yes	15(9,6)
					
**History of Previous Caesarean**	No	134(85,4)	**Complication during delivery**	No	149(94,9)
	Yes	23(14,6)		Yes	8(5,1)
					
**History of Previous complicated pregnancy**	No	146(93,0)	**Complication during postpartum**	No	150(95,5)
	Yes	11(7,0)		Yes	7(4,5)
					
**History of abortion**	No	114(72,6)	**The methods of feeding the baby by women**	Breast-feeding	114(72,6)
	Yes	43(27,4)		Bottle-feeding	21(13,4)
				Breast-feeding and bottle-feeding	22(14,0)
					
**High-risk Pregnancy**	No	99(63,1)	**Women's desires to continue thebreast-feeding**	Yes	136(86,6)
	Yes	58(36,9)		No	21(13,4)

**Figure 1:**
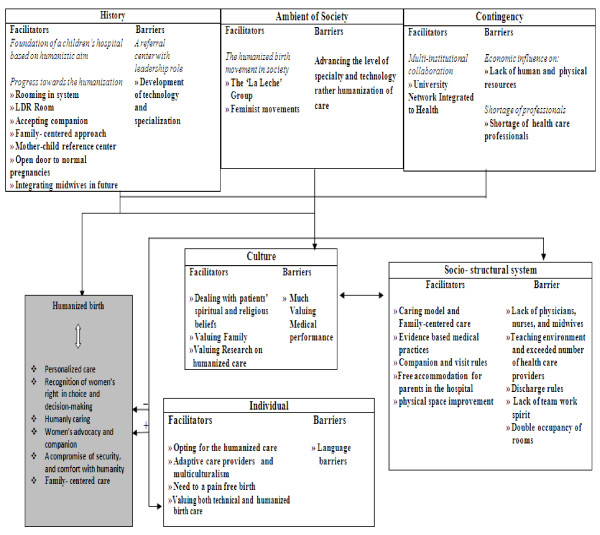
**Facilitator and barriers to humanized birth care in a highly specialized university affiliated hospital**.

The analysis of data consisted of two main general categories: the facilitating factors and the barriers. Sixteen themes and sub-themes emerged from the context describing the facilitating factors and eleven themes and sub-themes explained the barriers towards the humanization of birth approach in the studied highly specialized hospital. These themes and sub-themes are shown in details in Figure 1. The most important internal and external components of the institute acting as facilitators or barriers towards humanization of birth found in our analysis are shown in the following section:

### Internal Institutional Components: the structure, the individual, and the culture

#### The structure: As facilitating factors

##### ➢ Caring and family- centered model of care

The analysis of data showed that the hospital mission and its strategies concentrated on a *caring approach, based on the family's collaboration in the provision of care*. One of the hospital's prominent values was family. This value expresses the hospital's desire for the improvement of family's well-being, as well as its responsibilities towards the family unit (strategic plan 2007-2010). One of the nurse professionals mentioned that: "family is one of our big values; education is always related to the family." The hospital also valued 'respect', thought that it "must be reflected in the actions, attitudes, words and behavior of all employees, whether they are doctors, executives, or volunteers".

The women participant mentioned that they had the feeling that they were at the center of care: "it is not the caregiver who is at the center; it is really the child, the baby, the mother, and finally the patient who is at the center". The content analysis of documents also showed that the caring approach led to the creation of an environment in which the women had the opportunity to grow, learn, and adapt according to their own potential and experiences.

The findings from the questionnaires also showed that 80.9% of all women felt completely respected and accepted by the care providers. Most of them (47.8%) qualified the received care as a personalized or very personalized (42%) one, which was adapted to their needs (Table 2).

**Table 2:** Description of the quality of prenatal care in the highly specialized hospital

Criteria	N = 157	%
**The quality of follow-up during pregnancy**		
Very complete	97	61.8
Complete	57	36.3
Incomplete	3	1.9
**The care was**		
Very personalized	66	42
Personalized	75	47,8
Impersonalized	14	8,9
Very impersonalized	2	1,3
**Being in a good hand**		
Yes completely	128	81,5
Yes somewhat	26	16,6
Yes none or less	3	1,9
**Care provider was competent**		
Yes completely	138	87,9
Yes somewhat	17	10,8
Yes none or less	2	1,3
**Being respected and accepted by care provider**		
Yes completely	127	80,9
Yes somewhat	27	17,2
Yes none or less	3	1,9
**Being trusted by care providers**		
Yes completely	115	73,2
Yes somewhat	38	24,2
Yes none or less	4	2,5
**Encouraged by care provider **		
Yes completely	114	72,6
Yes somewhat	34	21,7
Yes none or less	6	3,8
Not really	2	1,3
**Satisfaction of care provider **		
Yes completely	123	78,3
Yes somewhat	28	17,8
Yes none or less	6	3,8

One of the administrators argued that their caring and family-centered philosophy, allows the family to act as a partner in care: "people are allowed to make informed decisions about their care, they make informed choices" (ADM2). From a total of 157 women, only 5.7% stated that they were not asked for their opinions, and 39 of 157 (24.8%) and 3 out of 10 interviewed women felt they did not participate in every decision that was made.

Nevertheless, analysis of the questionnaires showed that most of the women participants in questionnaire group (81.5%) and 9 out of 10 interviewed women were not allowed to choose between different birthing positions, nor eat during labour (86.6%) if so desired. One of women in the questionnaire group commented that she hid while she ate and it was frustrating not to be allowed to eat (QPID: 126).

##### ➢ Companionship and visiting rules

Almost all the women participants considered the companionship and visiting rules in the hospital as facilitating factors in the provision of humanized care. Analysis of the questionnaires showed that most of the women had had a companion present during prenatal visits (55%), as well as during labour and delivery (94.9%). Data from observation of deliveries showed that women could have as many companions in the LDR room as they chose. Most of the women (74.5%) in their questionnaires pointed that companion helped them a lot. The women participants affirmed that the humanization of birth is more prominent when the staff allows to have one's close relatives nearby, especially during medical interventions or operations. One of the low risk women who received epidural analgesia expressed her feelings about having a companion during the epidural intervention as follows: "I really had to hold on to somebody in order not to move... I was glad of the support I had at that time" (OB2). A high-risk woman expressed her feelings about the presence of a companion during a cesarean section as follows:

**OB 6: **It was reassuring to have someone there apart from all those people with their masks, their green coats, and caps [...] to have someone close, a family member, just to hold their hand" (high-risk woman).

Narratives from the women and the administrators also revealed that there was no real barrier concerning neither visiting hours in this hospital, nor the number of companion and visitors present. The questionnaire showed that most of the women (91%) could meet their companion whenever they wanted. A low risk woman stated that: "the staff was very receptive...there is never anyone who says anything" (OB10). Another woman said:

**OB 6: **Evening visits, for example; they are quite flexible here because they allow the family to visit at any time. Of course with some precautions [...] it is understandable that some parents cannot live without their child, or there are some who live far from the hospital, and cannot come earlier during visiting hours (a high-risk woman).

A hostel-like service in the hospital, accommodated parents for a week after the mother's discharge without any extra charge. Administrators and professionals considered the presence of hostel as a facilitator for humanized birth care because it "permitted parents to be hosted in the hospital and in proximity to their sick child (Prof4) and mother could breastfeed her baby on demand" (ADM3).

#### The structure: As barriers

##### ➢ Lack of spirits of communication among professionals

Nurse professionals saw the lack of communication between professionals as an important barrier in the provision of more humanized birth care. The nurses pointed to the lack of good communication between professionals in childbirth and postpartum units. One of the nurses said that 'they seem like two different worlds' (Prof3). Another nurse said that "communication is not always clear between the nurses, we are not all aware of the history of the patient" (Prof5). She also mentioned that the lack of communication could be stressful: "sometimes the night shift person leaves and when we arrive, there is no report" (Prof5). Most of the nurses agreed on a lack of good communication between nurses and physicians.

The nurse professionals also mentioned that the overload of work prevents all the professionals from establishing a good level of communication; an administrator, in fact, said: 'the barrier is actually the workload. Everybody runs, and everybody works hard' (ADM3). Many of the women participants complained (in the questionnaire) about the lack of communication between their health care providers and said: "I'm complaining about the lack of communication between the units. When I arrived, my documents were still in the medical records department"; "during a change in shift, they did not bring me my daughter for breastfeeding, as they did not know they had to"; "the communication between nurses during the changing of the shift was sometimes bad"; "communication between night and day shifts should be improved."

##### ➢ Teaching Environment

Analysis of data from the interviews, observations, field notes, and the self-administered questionnaire, showed that a teaching environment, and the presence of a large amount of health care professionals, can be considered as a barrier for humanized birth care as it can interfere with women's privacy as well as their families', a lack of intimacies, and a lack of continuity of care. Descriptive analysis of the questionnaires showed that some of the women (42.7%) had three to four care providers present during labour and delivery, and that about 24% of the women had five or more care providers at this time. One of the obstetricians stated that:

**Prof 9: **we have a teaching environment. So we have the externs, the residents, the interns, also physicians and nurses, etc. and this is unfortunately a barrier to the humanization of care, because, for a patient, we are compelled to say her: "well, listen, we are in an academic environment, so for your delivery, there will not only be three doctors there, who are not necessarily yours, but the doctor in shift, and also a resident and also an extern, and perhaps also interns and nurses. It is unfortunately a bit contrary to humanization, but we have a duty to expose our students and residents in training, so that's a barrier: the educational environment (obstetrician).

Most of the women participants qualified the number of care providers present fairly (67.5%), and some qualified it too high (12.1%). Only 22 (14%) of the women had, during delivery, the same care provider who followed them during pregnancy, but even this came just for the birth of the baby. About 29.3% of the women said that it bothered them a little, not having their care provider with them during labour and delivery.

A high-risk pregnant woman said that: "a teaching hospital could be a disadvantage to humanized birth" (OB8), and another woman in questionnaire group commented: "we were disturbed a lot by the presence of a lot of care providers that did not consider our comfort" (QP: 152).

A nurse professional talked about night shift students who do not always respect the families' rest and sleep. (Prof 5)

The professionals' concerns were mainly about the dignity and privacy of women, and they stated that the environment constantly undermines their efforts for humanized birth, thus making it difficult for them to keep their calm. One of the obstetricians stated: "while nurses should talk to women who are going through a difficult time, with everyone entering the room constantly, it's impossible to keep track of the women" (Prof8). She continued that the presence of students could be a constraint as it could be preventing them from having better contact with their patients (Prof8). An administrator mentioned that in high-risk pregnancy cases, the number of health care professionals is even higher. "When women are at high-risk, they become an interesting case to more residents, and there are more doctors who go to see them" (Adm6). Moreover, the analysis of data from observation and interviews showed that the frequent rotation of students and trainees was bothersome for women and their families, as information about the women would have been asked many times.

##### ➢ Discharge rules and physical environment restrictions

Analysis of documentation from field notes and interviews showed that some mothers are urged to leave the hospital even if they are not psychologically and physically prepared, or have not received enough information. The nurses were generally in agreement that this discharge rule was a barrier to the humanization of hospital birth. One of the nurse professionals stated that: "some days you have to send the mother home. Everybody says: fast, fast, fast, and it's over. I'm sorry, but we also think this is inhumane" (Prof3). Another nurse said that:

**Prof 1: **when we get a surplus number of births compared to the number of beds, our response may take away a little bit from the humanized care approach. Sometimes the discharges are signed for mothers, even if they are not necessarily ready to go. At this point, I do not feel very humane when I tell mothers: I'm going to pack your stuff up and take you to the front door (nurse).

The pediatrician's interview revealed that the early discharge rules come from the hospital's administration. She stated that: "administrators think in this way: do not occupy beds unnecessarily! They force us to sign a discharge after 48 hours, before twelve noon" (Prof11). The nurse professionals talked about their experiences with mothers who were made to leave their rooms and were temporarily accommodated in 'the hospital while their children remained in neonatal care for medical reasons. "Mothers are crying and the parents are often split because of this situation" (Prof3)

Some parturient women had to share a room with another parturient woman in the postpartum unit due to the lack of sufficient financial support. Many of the women found the double rooms to be very inhumane, as well as uncomfortable. There was little space in the rooms, and it is always noisy and generally crowded. The women also complained about not being able to sleep at night. Most of the husbands also had difficulties staying with the mother at nights, since there was no space in the rooms to put a bed up for them. Moreover, the mothers felt that the double room put them in an uncomfortable situation. One woman stated that: "I am not a practitioner of religion, but I do not like to sleep in a room where there is another male companion present (QP: 148). One of the women participants explained that she had to change her room from a common room to a private one, since: 'there was really such a chill there and there was no intimacy whatsoever' (OB7).

A nurse professional mentioned that the rooms, which were shared between two women, were providing the contrary of humanized care, as they were the contrary of family-oriented care and intimacy: "it's just a curtain separating the two people" (prof3), "we cannot talk loudly because we must respect the patient's confidentiality" (Prof5). The nurse professionals also had difficulty providing nursing care for women and babies in the double rooms: "there is not much space to bathe the baby, so we cannot get the nursery" (Prof5). The administrators emphasized that in the humanization of birth model, your baby is supposed to be close to you, while in this case, the care providers are forced to take care of the babies in the nursery, since there is not enough space in the postpartum rooms. (ADM6, ADM2).

#### The individual: As facilitating factors

##### ➢ Needs to have a pain-free birth

Further analysis of observations and field notes revealed that women needed to have the option of a completely pain-free labour and delivery. Almost 43% of women during prenatal care were found to be afraid of giving birth completely or somewhat, and some of the women felt would not be able to control pain none or less (26.1%)/or not really (14%). However, the analysis of the questions related to the women's feeling during labour showed that about 20% of the women felt not powerless at all or felt only somewhat powerless (28%) while many of the women were not sure about their feeling of power (28.7%) or control (29.3%) during labour. As a whole, 95 out of 157 (60% ) of women (7 out of 10 interviewed women) had received epidural analgesia during labour, while most of them had used other methods of relieving pain, such as: medication, walking, changing position, breathing, and showering, before deciding to have the epidural analgesia. Even though many of the women experienced some mild side effects from the epidural analgesia, such as: dizziness, lack of control during contractions, and consequently, perineal lacerations; they stated that they were not disappointed with this method of pain relief. Most of the women participants in fact stated that they were satisfied with their painless childbirth experience, and that they found it to be a humanistic approach to birth. One of women said that: "I demanded pain relief at my first, second, third deliveries" (OB9). Descriptive analysis of data from the questionnaires showed that women who felt themselves powerless and those who did not, received both epidural analgesia.

**OB 10: **It was my decision to have it as soon as possible. I was not for, or against it. I said, I will see how I feel... but when I started to suffer, I wanted to have the epidural, and I had a super nice delivery (low risk woman).

**OB 9: **I would not be able for a normal delivery, I am not capable. Without an epidural, I would not be capable. I am afraid of pain, I do not like pain, I would never be able (woman at low risk).

The professional anesthetist believed that the epidural is a convenient factor for mothers, and a way to make birth more humanized:

**Prof 10: **There are some people who say that an epidural (analgesia) is not humanized because it is not natural; it is an invasive technique in a certain way. But it is true that removing pain helps the woman, perhaps it makes her more ready to handle her baby (anesthetist).

##### ➢ Valuing of both technical and humanized model of childbirth

Analysis of data from interviews, observations, and field notes, showed that the staff and women valued both technical and humanized model of care at birth, which is tailored to improve safety, assurance, and comfort for women and their children.

One of the obstetricians stated that the medical intervention does not exclude humanized care for him and he is doing all monitoring and medical intervention very humanely (Prof8). The administrators seemed to show an interest, in adapting different interventional protocols with the humanistic approach. Most of the nurse professionals expressed their willingness to provide a more personalized and humanized kind of care, in spite of the shortage of nurses, and the overburden of work and responsibility in the hospital. The pediatrician who was interviewed pointed out that even in the midst of a shortage of nurses, these still tried to provide a gentle and humane approach to the parents (Prof 11). A nurse also stated that they had excellent doctors and nurses in this hospital, who do 'the best they can'. However, "it's mainly the nurses who care about the humanization of care; and the ones who are the most sensitive to the client's needs" (Prof 4).

The interviewed women valued both the medical and specialization as well as humanized aspects of care:

**OB 3: **The fact that this is a specialized kind of care and is thus medically driven was okay for me [...] I was expecting something humane as well as high levels of medical technology where no errors are committed if handled with serious and meticulous care. Maybe that can't be found anywhere else than here (low risk pregnancy).

Our findings showed that 62 (39.5%) of all157 women (5 out of 10 interviewed women) were completely or somewhat ready for some kind of medical interventions; and only 12 out of the 157 disagreed with the medical intervention they received. None of the interviewed women disagreed with the perinatal medical interventions.

The findings also showed that 98.1% of women had an EFM. With the exception of 2 out of the 157 women, all the women felt that they were safe, and that they had a competent care provider which was able to handle any unpredictable problem. One of the women interviewed stated that: "you feel a special care here, the staffs are very competent; otherwise, they might not have positions in a hospital like this, especially during deliveries" (OB2). All of the women participant but 3 felt that they were in good hands. One of the women, who was pregnant with twins, said that:

**OB 4: **I personally preferred to have access to all available care in case an urgent situation raised. I felt more confident coming here than going into a home birth (high-risk woman).

About 95% of women- including all of the interviewed women- were satisfied with the care they received themselves, and that given to their babies. Most of the 157 women (56.1%) and 9 out of 10 interviewed women answered that their delivery went better than they expected. Except for 9 out of the 157 (1 out of 10 interviewed women), all the women said that they would choose the same setting for their next pregnancy if there would be any place for them. The common reasons expressed by the women participants in the questionnaires for choosing this hospital were: satisfaction with care, competence of the care providers, and the sense of assurance and security felt by giving birth in a highly specialized hospital for children.

#### Culture: As facilitator

##### ➢ Dealing with patients' spiritual and religious beliefs

The administrators placed an emphasis on perinatal mourning, where they said they were confronted with different cultures as well as religions, and stated that the hospital was well adapted to the practices and different cultural beliefs of mourning. The administrators also stated that it was interesting that a hospital with a French-Catholic root had adapted its services for all kinds of cultures and religions:

**ADM 4: **We have a program of mourning. We train our professionals to be open to all kinds of cultural or religious reactions which they might be confronted with. We do all we can really do, in order to provide them with choices with which to deal with their deceased baby as they see it. Some people do not bring a priest, but bring someone from their religious practice instead, and they all gather in a room. We always try to adapt our interventions in concordance with the cultural or religious beliefs of the family under question.

The interviewed professionals and administrators mentioned that this hospital's customer profile had changed a lot, and that this had led to certain services having to be adapted. One of the administrators stated that the hospital offered spiritual support: "In the past, we had a pastoral service that was rather based on needs related to the Catholic religion, but now we no longer talk of religion" (ADM4). One of the women said: "I had the privilege of being treated with no mention of my religion... everything was organized clearly, and due respect was paid to patients"( OB2).

#### Culture: As barriers

##### ➢ Valuing Medical Performance

Many of the administrator participants argued that the culture of care around high-risk pregnancies in specialized hospitals, and the highly esteemed medical aspects of this care, both act as barriers to the humanization of birth. One of the administrators stated the following: "this is a tertiary hospital, so we expect to have high-risk pregnancies, and babies that are the highest at risk. Everything is pointing in this direction" (ADM2). The administrators also agreed that working in a tertiary center meant resources which allowed for medical specialization, as well as skilled training.

The opportunities for the development of expertise present in this environment lead the physician to gravitate more quickly towards medical intervention. An administrator stated that: "there is no place for an unspecialized professional in high-risk pregnancies or for midwives who are trained for normal pregnancies" (ADM5). The obstetricians argued that "they are valued for their medical performance, not for the fact that they listen to their patients, or because they spend time with them" (Prof8). They also argued that the hospital had been upgraded, and is valued by its effectiveness in having reduced waiting time in the emergency room and caesarean section rates, as well as increasing survival rates. This was not done for the humanization of care:

**Prof 8: **No-one gives us an assessment at the end of the month and asks us to look at ourselves and our patients and see where we have been humane...I'm told: 'you've had so many deliveries, and your forceps rate is this, and your cesarean rate is that. At that point we are evaluated and compared ... The indicators of good performance are always expressed in terms of the number of patients, number of births, number of emergency room visits, and the number of new cases being visited. This is rarely calculated on a measure of the humanization of care (obstetrician).

### External Institutional Components: the contingency, the history, the society

The most important facilitating factors and barriers observed in the external environment of the hospital were related to its contingency.

#### The contingency: As facilitating factor

##### ➢ Multi-institutional collaboration

The specialized hospital under study was an integral part of an Integrated Health University Networks (RUIS: Réseau Universitaire Intégré de Santé) in Montreal. Working in a network has improved the *quality, accessibility, and continuity *of care to mother and child, as well has increased access for women and their families to advanced technologies, information, and promoted the harmonization of care practices. Moreover, one of the nurse professionals argued that working in a network helped to maintain the continuity of care, as well to increase the continuity of information to provide to the mothers (Prof1). Another nurse mentioned:

**Prof 2: **We have already taken a step towards the humanization of birth. We have patients who prefer to be visited at home. There exists home care for high-risk pregnancies. They live in their environment, and we can provide good quality of care for them (nurse).

#### The contingency: As barriers

##### ➢ Shortage of professionals

General shortages of professionals lead to a lack of choice of a health care provider or a place of birth by women. One of the obstetricians said that: "it is sometimes a challenge to ensure the presence of a physician in a certain environment at the time of delivery" (prof7). An administrator said that many young mothers were not able to meet a doctor during the first 20 weeks of their pregnancy, even though many of them sought a doctor at this hospital; the hospital simply could give them an appointment (ADM6). A descriptive analysis of the data collected from the questionnaires showed that only 30 out of 157 (19.1%) had a choice of the care provider, and 7 out of the total of 157 women (4.5%) chose the hospital themselves; and that all the others were admitted by chance depending on the availability of the physicians.

However, almost all of the women interviewees were aware of the hospital 's reputation. Many of women, including those at high-risk, declared that they had difficulty in finding a physician. The women participants in the questionnaire group responded to the question of why they did not choose a care provider themselves, as following: "we could not choose because there were not enough doctors", "I was looking for a doctor and this doctor was the one available", "It was controlled by hospital policy", or "I was referred to this doctor". One of the high-obstetric-risk women stated that: "I was not expecting to be followed at this hospital; there were so many requests, so I was very lucky to be followed here" (high-risk woman). Further descriptive analysis of the questionnaires also showed that about 93% of women received care from obstetricians and gynecologists (33.8% male, 59.2% female), 3.8% from family physicians, and 2.5% were joint care providers. None of the deliveries were assisted by midwives in the hospital.

The lack of family doctors, specialist nurses, midwives, and psychotherapists in the hospital, was considered as a barrier to humanized birth care by almost all of the interviewed participants. One of the women participants remarked in the questionnaire: "pregnancy is not sickness, the midwife should be present in the hospitals" (QP: 70). An obstetrician professional argued about the importance of removing specialists from normal pregnancies, and replacing them with midwives in the future (Prof9). One of the administrators who agreed with the presence of midwifery professional at hospital stated that: "I do not think we always need to have specialists in order to deal with people who have a normal health status" (ADM1).

Almost all of the administrators and professionals agreed that preventing work overload by hiring more professionals can help in the humanization of birth care. The administrators argued that they needed more time to be able to provide that kind of humanized care: "the shortage of personnel causes work overload and stress, which in turn raises tiredness; and when you're not well yourself, it makes it hard to heal others" (ADM3).

Lack of necessary financial support from outside sources considered as a barrier to humanized care in the studied hospital. One of the obstetrician professionals stated that the health care system is in shortage of money which leads to establishing priorities and that lack of financial support by the government has forced the hospital not to place the issue of humanization as a top priority (Prof7). Obstetricians' narratives also revealed that, in spite of the fact that most of money in the hospital has been invested on the physical security of the patient, while the investment on the psychological aspects of birth care - a factor which has led to the reduction of the perinatal mortality rates in Canada to the lowest in the world- has in fact been ignored:

**Prof 8: **We are talking about high-risk patients who are faced with losing a child, or losing a pregnancy. There are very few psychological (support) resources in the hospital, so we cannot deal with these patients properly. This is a major obstacle to the humanization of care for these women (obstetrician).

## Discussion

Our findings showed that many of the components of the external and the internal environment of a highly specialized hospital can act as facilitating or barriers for the 'humanization of birth' approach.

To summarize, our findings showed that most of the high-risk and low risk women were generally satisfied with the care and services they received in the highly specialized hospital and they would return back to the same hospital if they had a choice. Our findings are similar to those of De Koninck et al (2001), in that approximately 88.5% of physicians' clients of in Quebec hospitals indicated that they wished to deliver in the same setting for birthing if they became pregnant again [22].

One of the facilitating factors of the humanized birth practice in this highly specialized hospital was seen to be the hospital philosophy, and strategies, which had been founded on family-centered care. The family-centered care approach of this hospital had already opened a door for professionals to share responsibilities with their patients, whilst still caring for their health. Our findings showed that women and families in this hospital were respected, and received a personalized kind of care. Previous research had shown that many women, who were looking for a midwife caretaker, were concerned about the 'individual' or 'personalized' and 'family-centered' aspects of care. In Parry's study, women discussed the importance of their husband's involvement in their childbirth; and they expressed their feelings that their husband wouldn't have been nearly as involved if they hadn't had midwives [27]. However, the findings of our study revealed that integrating family involvement, and providing family-centered care, is also achievable in a highly specialized hospital, and that this was in fact a facilitating factor for the humanization of birth in such a context.

In almost all of the reviewed literature, the humanization of birth is defined as the use of decreasing levels of medical intervention in the normal delivery process (Brunt, 2005; Davis-Floyd, 2001; Page, 2000). In contrast to this, the humanization of birth in a highly specialized hospital is not, however, perceived in this way. None of the low or high-risk women in our study, however, complained about the medical and technical care provided to them; and on the contrary, they found it to be a necessary element of a secure birth. None of the women expected the care providers to respect their bodies' physiologic capacity in giving birth without medical intervention. We have learned from the findings of this study that even though most of the women interviewed reported the positive experiences of childbirth, women in a highly specialized hospital are increasingly being faced with the medicalization of birth. The women participants valued technology and the specialization of care, and even considered it as a facilitating factor for the humanization of birth, as it brought them reassurance and comfort. It was clear for women that a highly specialized hospital had its own frame of reference or 'language', and a highly technical one, and women and their families acted in accordance with the values and technologies surrounding them. On the other hand, women and their chosen hospital had the same codes and language in care. In our study, almost all of the women participants expressed no concerns about a natural birth. This contrasts with women who chose midwives or birth attendants as their care providers and who gave birth in a birthing centre. These women exhibited a resistance to the medicalization of birth, and opted for a natural birth, as well as seeked for continuity of care [27].

Moreover, our findings showed that women had an increased tendency to want to give birth in a specialized children's hospital, as they saw it as being the best place for the safety and security for their baby. This result was similar to one of the findings of Jimenez's study which revealed that for many of the women who chose hospital, it remained "the ultimate safe place to bring a child into the world"[28]. Cindoglu's 2010 study also showed that almost all Turkish women opted for medicalization due to their concern for a safe birth [14]. Our findings also was similar to the study of De koninck et al (2001), where safety was considered as an important criterion for the quality of care for physicians' clients at hospitals and many women said that "if something goes wrong, we are in the right place"[22]. Hausman argued that the way birth is defined as a risky event, leads to the over use of medical intervention and technology by physicians, even in the case of normal births [29]. The medicalization of birth has come about due to the view that pregnancy as a time of risk and danger for the woman [30]. The women who prefer technology and who rely on medicine and obstetrics, are more likely to consider the medicalization of birth as a means of reassurance, a reflection of the technological society, or finally as a result of fear as to the outcome of their birth [31]. Henly-Einion has recently argued that "the concept of choice does not appear to be between natural and interventional birth, but between normal medical labour and complicated medical labour" [31].

Most of the participating women in the study felt that they could not go through labour and give birth whilst controlling their own pain. Thus, they requested an epidural analgesia in order that they may have a pain-free birth. Women found that epidural analgesia was a facilitating factor in the humanization of birth care. Our findings also showed that the presence of a companion and the emotional support provided by this companion, as well as the use of other methods of relieving of pain -such as massages and breathing- did not change women's decision to have an epidural. Noticeably, during the data collection period, there had been no whirlpool baths available in the hospital; however, during the last field visit to the hospital, this method of relieving pain was seen to be provided for the women. Nevertheless, most women still requested epidural analgesia for pain relief. Paradoxically, in Parry's 2008 study, it was found that the Canadian women who chose a midwife felt they were more empowered than ever, and that they had full control over their bodies. Comparing women's quotes from Parry's study: "I just get the feeling that I can do this, and it's really not that big of a deal" [27], with the quotations of women from our study "I would not be able to deal with for a normal delivery, I am not capable. Without an epidural, I would not be capable of doing it; I am afraid of pain, I do not like pain, I would never be able", clearly shows the individual differences on these issues, as well as the variety of women observed in society, some of whom seek midwifery care, and some who choose highly specialized hospitals.

The literature indicates that women's fear of pain at birth is depended on how women are prepared for birthing during prenatal care or even how they are informed about it by surrounding people. Empowerment at childbirth is relevant to midwifery care as the support of midwives is one of the most fundamental factors in a positive childbirth experience and help women to being in control of their body, mind and choices. The lack of support and understanding for the fear among those who provide care during the prenatal period and lack of enough information about the physiology of pain make women more dis-empowered [32]. Melender's study (2002) showed that elements like previous experience, knowledge, or uncertainty caused fear to be associated with childbirth. Having knowledge found to be a very important means of removing or alleviating fear (Melender, 2002). The women participants in our study received information regarding pregnancy and childbirth through different meetings and prenatal classes, but it seems that this information was not sufficient or supportive enough to overcome women's fears about birthing. In order to alleviate the fear of childbirth, and the feelings of loss of control experienced by women during labour and delivery, health professionals should focus on empowerment strategies, as well as preparing women for labour during prenatal visits, or even before their pregnancy. This would help women regain control over their bodies, reduce the level of distress they experience during labour and delivery, and thus avoid the overuse of medical interventions in birth, such as epidural analgesia, and cesarean sections.

The findings also showed that professionals and administrators in the highly specialized hospitals valued the humanization of birth, and were proud the reconciliation of medical intervention and humanistic approaches to care. The attitudes of maternity care professionals, means nurses, obstetricians and pediatricians towards childbirth practice were not limited to providing optimal care through the use of obstetric technology, but to provide both physical and psychological care for women and their families. The humanization of care could be achieved through the validation of human beings, and one step towards this is "allying technical and humane competencies in professional practices" [33].

Our findings revealed that changes have been made -or are going to be made- to the physical environment of the hospital and the maternity wards, in order to prepare for its evolution into a natural birthing centre, as well as to provide a more pleasant environment for women and their families during their hospital stays. Nevertheless, there were also still many barriers present, and these included women's choice limitations, lack of good communication between professionals in different units of the maternity ward, and lack of communication between professionals in different work shifts and finally the presence of a lot of health care professionals raised questions on the issue of privacy and dignity, and continuity of care; then, these were also considered barriers for the implementation of a more humanized birth care approach. The finding of a recent Canadian Perinatal Survey achieved by Maternity Experiences Survey (MES), revealed that only one-half (49.4%) of Canadian women had received continuous care in term of support from the same provider during pregnancy and at birth, while most of the women (88.4%) believed that it was important to have the same provider [34]. This is imperative if the stakeholders in health care system are to attempt to ease the present overload of work, and provide continuity of care ranging from the women's first antenatal visit to home visits after birth as well as offering psychological and emotional support to women. The collaboration between the Centre de Santé et de Services Sociaux (CSSS) that midwives are part of it, and hospital centres guarantees that not only the women receive continued care, but also they would have access to different services and professionals in hospitals. This is what will enhance their sense of security.

Mota et al. (2006) stated that the humanization of care should be constituted as a policy in the organization of the health care system, based on the principles and modes of relationships between professionals and clients, and between the different professionals and different units of the health care services [35]. According to the national humanization policy in Brazil, humanization involves knowledge transfer between the health care providers and clients, as well as between professionals and the ways their teams work together [36].

### The strength and limitation of the study

Using a mixed quantitative and qualitative method of collecting data, and the collection of an excellent variation of samples provided a rich pool of data for this study. The interpretations of the findings are shaped on the basis of triangulation of four sources of data, as well as of our in-depth knowledge in this field. However, this study, as any other, has some limits. The findings cannot uncover whether these were the women's culture, and/or the culture of birth place, or if the availability of obstetric technology, and the easy access to epidural analgesia - which is covered with insurance policies- that resulted in the high rate of demand for epidural analgesia observed in the studied hospital.

We tried to describe the research methodology including sampling, methods, and analysis in detail, which was used - to increase the transferability of the findings. The nature of this study, however, does not allow generalizing findings, as they do not reflect the practices of all obstetrics departments, in all highly specialized hospitals, regarding the humanized birth care issue in the province of Quebec in Canada. The level of obstetric interventions in different hospitals could change according to the hospital's mission, the level of care offered in that setting, and the characteristics of its target population.

For future research on this topic, we suggest a comparison of the facilitating factors and barriers towards humanized birth in the highly specialized hospitals, in different countries, where, the culture of childbirth is different from what we experienced in Canada. The setting of the highly specialized hospitals should be examined further for the feasibility of introducing more options for women, and for their right to make choices, if it aims at improving the practice of humanized birth care. More research should thus be conducted in order to understand what options and choices are realistically available to pregnant women who come to a highly specialized hospital to give birth to their child, as well as the factors which women take into account when making these choices if there is a possibility for it.

## Conclusion

The implementation of the humanization of birth practices in the highly specialized hospitals aims at making the experience of hospitalization more reassuring, comfortable, and pleasant, for women and their families. A high level of technology and expertise, as well as caring, family-centered and continuity of care are all necessary to ensure the provision of humanized care in such an institution. The studied highly specialized hospital considered a safe place for women and their child in the case of a need for immediate access to medical care and technology. The argument of medical intervention and technology at birth being an opposing factor to the humanization of birth was not seen to be an issue in the studied highly specialized university affiliated hospital. Providing a pain free birth and technical care in a humane manner is essential to cover the humanized aspects of childbirth care and ensuring the satisfaction of women and their families who seek care in a highly specialized hospital. From the finding of this study authors conclude that mothers, children and families must benefit of progress in obstetric technology, but still a balance between security and humanity is essential.

When the aim is to improve the humanization of birth care in the highly specialized hospitals, the question of educating more health care professionals and integrating more care providers, especially midwifery and psychiatric professionals needs to be addressed by the stakeholders in health care system and hospital administrators. The greatest distress exhibited by women in this studied setting, was due to their hopelessness in having a guaranteed place for delivery before the onset of labour. This is imperative if the stakeholders in health care system are to attempt to ease the present overload of work, and provide continuity of care ranging from the women's first antenatal visit to home visits after birth as well as offering psychological and emotional support to women.

## Competing interests

The authors declare that they have no competing interests.

## Authors' contributions

Four persons have fulfilled the conditions required for authorship. Author 1(RB) has coordinated the paper from writing its protocol, taking approvals, designing the semi-structured questionnaires, collecting the data, transcriptions, analysis, and redaction of the manuscript. Author 2(MH) supervised the project from beginning to the end, helped in implementing the research, helped in qualitative analysis and validate the methodology, and participated in drafting the manuscript. Author 3(LG) also supervised the project, participated in the design of the study and questionnaire development. Author 4(WF) helped in preparing the field of research and participated in drafting the manuscript. All authors read and approved the final manuscript.

## Pre-publication history

The pre-publication history for this paper can be accessed here:

http://www.biomedcentral.com/1472-6874/11/53/prepub
